# Highly focused anopheline breeding sites and malaria transmission in Dakar

**DOI:** 10.1186/1475-2875-8-138

**Published:** 2009-06-24

**Authors:** Vanessa Machault, Libasse Gadiaga, Cécile Vignolles, Fanny Jarjaval, Samia Bouzid, Cheikh Sokhna, Jean-Pierre Lacaux, Jean-François Trape, Christophe Rogier, Frédéric Pagès

**Affiliations:** 1 Unité d'entomologie médicale, Equipe 7 "Maladies émergentes et moustiques", Institut de Médecine Tropicale du Service de Santé des Armées, Allée du Médecin colonel Jamot, Parc du Pharo, BP60109, 13262 Marseille cedex 07, France; 2 Unité de Recherche sur les Maladies Infectieuses et Tropicales Emergentes – URMITE – UMR6236, Institut de Médecine Tropicale du Service de Santé des Armées, Allée du Médecin colonel Jamot, Parc du Pharo, BP60109, 13262 Marseille cedex 07, France; 3 Unité de recherche en biologie et épidémiologie parasitaires, Equipe 7 "Maladies émergentes et moustiques", Institut de Médecine Tropicale du Service de Santé des Armées, Allée du Médecin colonel Jamot, Parc du Pharo, BP60109, 13262 Marseille cedex 07, France; 4 Unité de Recherche sur les Maladies Infectieuses et Tropicales Emergentes – URMITE – UMR6236, Institut de Médecine Tropicale du Service de Santé des Armées, Allée du Médecin colonel Jamot, Parc du Pharo, BP60109, 13262 Marseille cedex 07, France; 5 Centre National d'Etudes Spatiales – Application Valorisation – 18 avenue Edouard Belin, 31401 Toulouse Cedex 9, France; 6 Observatoire Midi-Pyrénées, Université Paul Sabatier, 14 avenue Edouard Belin, 31400 Toulouse, France; 7 Unité de Paludologie Afrotropicale, Equipe 7 "Maladies émergentes et moustiques", Institut de Recherche pour le Développement, Route des Pères Maristes, BP 1386, 18524 Dakar, Sénégal; 8 Unité de Recherche sur les Maladies Infectieuses et Tropicales Emergentes – URMITE – UMR6236, Institut de Recherche pour le Développement, Route des Pères Maristes, BP 1386, 18524 Dakar, Sénégal

## Abstract

**Background:**

Urbanization has a great impact on the composition of the vector system and malaria transmission dynamics. In Dakar, some malaria cases are autochthonous but parasite rates and incidences of clinical malaria attacks have been recorded at low levels. Ecological heterogeneity of malaria transmission was investigated in Dakar, in order to characterize the *Anopheles *breeding sites in the city and to study the dynamics of larval density and adult aggressiveness in ten characteristically different urban areas.

**Methods:**

Ten study areas were sampled in Dakar and Pikine. Mosquitoes were collected by human landing collection during four nights in each area (120 person-nights). The *Plasmodium falciparum *circumsporozoite (CSP) index was measured by ELISA and the entomological inoculation rates (EIR) were calculated. Open water collections in the study areas were monitored weekly for physico-chemical characterization and the presence of anopheline larvae. Adult mosquitoes and hatched larvae were identified morphologically and by molecular methods.

**Results:**

In September-October 2007, 19,451 adult mosquitoes were caught among which, 1,101 were *Anopheles gambiae s.l*. The Human Biting Rate ranged from 0.1 bites per person per night in Yoff Village to 43.7 in Almadies. Seven out of 1,101 *An. gambiae s.l*. were found to be positive for *P. falciparum *(CSP index = 0.64%). EIR ranged from 0 infected bites per person per year in Yoff Village to 16.8 in Almadies. The *An*. *gambiae *complex population was composed of *Anopheles arabiensis *(94.8%) and *Anopheles melas *(5.2%). None of the *An. melas *were infected with *P. falciparum*. Of the 54 water collection sites monitored, 33 (61.1%) served as anopheline breeding sites on at least one observation. No *An*. *melas *was identified among the larval samples. Some physico-chemical characteristics of water bodies were associated with the presence/absence of anopheline larvae and with larval density. A very close parallel between larval and adult densities was found in six of the ten study areas.

**Conclusion:**

The results provide evidence of malaria transmission in downtown Dakar and its surrounding suburbs. Spatial heterogeneity of human biting rates was very marked and malaria transmission was highly focal. In Dakar, mean figures for transmission would not provide a comprehensive picture of the entomological situation; risk evaluation should therefore be undertaken on a small scale.

## Background

### Malaria and urbanization

Urbanization has a significant impact on the health of local populations. It is estimated that by 2025, 800 million people will live in African cities and urban malaria is considered to be an emerging health problem of major importance in Africa. Urban malaria should be seen as a specific public health issue and assessment, understanding and control should not simply reproduce initiatives taken in rural communities [1,2].

In urban settings, malaria risk heterogeneity is recorded over small distances due to diversity in the degree and type of urbanization, density of human population, quality of water and waste management, vector control measures, household factors and access to health care [1,3], or human migration patterns that might import parasites from rural areas [4]. Urbanization has a great impact on the composition of the vector system and malaria transmission dynamics [5]. In regard to breeding requirements, there is evidence of adaptation of anopheline species to urban settings and several examples of polluted breeding habitats or new types of breeding habitats have been brought to light [6-9]. The importance of urban agricultural activity on malaria has also been reported in several African cities, such as in Côte d'Ivoire and Ghana [10], where irrigation leads to the creation of larval habitats [10,11] and higher malaria prevalence [12,13].

Finally, variations in *Anopheles *densities play a major role in the spatial and temporal heterogeneity of malaria risk. In cities, where blood meal sources are abundant, dispersion of the vectors is low and malaria transmission is focal and highly driven by the proximity of breeding sites [14,15]. Thus, an understanding of transmission heterogeneity requires a good knowledge of the geographical localization of breeding sites. Characterizing and mapping these habitats will help to spatially rank malaria risk in urban settings and focus control activities on a small scale [16].

### Clinical malaria in Dakar

In Dakar, the capital city of Senegal, some malaria cases are recognized to be autochthonous [17] but parasite rates and incidences of clinical malaria attacks in the city and its nearby periphery have been recorded at low levels compared to continent-wide level [14,17,18]. Nevertheless, malaria should not be neglected, as severe cases have been reported among Dakar residents with little acquired malaria immunity [19]. In some health facilities, up to 65% of patients diagnosed with malaria present severe forms of the disease [20]. In Dakar, a high prevalence of severe anaemia was found in young children between 1990 and 1996 [21] and placental malaria infections have been associated with pre-eclampsia in pregnant women with poor malaria immunity [22]. In the nearby suburbs, it has been found that 10% of delivering women were positive for *Plasmodium *parasites in the placenta and 44% of placentas showed chronic infection, associated with low birth weight [23].

### Malaria transmission in Dakar

In this clinical context, local malaria transmission has been studied for several decades. In Pikine, a suburban area of Dakar, transmission was demonstrated in 1979–80, with anopheline aggressiveness peaking at more than 100 *Anopheles arabiensis *bites per person per night (*Plasmodium falciparum *sporozoïte rate up to 1.14%) and an Entomological Inoculation Rate (EIR) of 43 infective bites per person per year [24]. Less than 10 years later in the same city, *An. arabiensis *was still the main anopheline species captured but the estimated annual EIR did not exceed 0.382 [14].

In the south and central sanitary districts of Dakar, in 1994–95 and 1996–97 respectively, *An. arabiensis *aggressiveness was low, with less than one bite per person per night and no infected *Anopheles *collected [17,25]. In 2005–2006, malaria transmission was assessed in two vegetated areas of downtown Dakar during the wintering periods; the recorded aggressiveness peak was close to 200 bites per person per night and the EIR was up to 9.5 infective bites per year [26].

The results underline possible changes in the entomological situation in the Dakar region and suggest the need for larger entomological investigations, in order to assess the current malaria transmission risk in the area.

### Breeding sites

Every *Anopheles *species has its preferred water bodies for oviposition, depending on climate, physical geography and human activities. Breeding sites can be natural or man-made, of various sizes, located in running or stagnant waters, shaded or sunny, permanent or temporary.

The main anopheline species found in the Cap-Vert peninsula are members of the *Anopheles gambiae *complex. *Anopheles arabiensis *is the major malaria vector and usually breeds in small, temporary, clear and shallow water, with small amounts of organic matter and surface vegetation [27]. In 2005, the following species were also found in Dakar [26]: *Anopheles melas*, a salt-water species, and one specimen of *An. gambiae s.s*.

In and around Dakar, temporary breeding sites can appear during the rainy season in tyres, step tracks, puddles, ditches and garbage cans, or in debris on construction sites. Anopheline larvae have also been sampled in permanent water collection sites, such as permanent swamps created by the rise of the water table, which are known locally as "niaye" [14], or permanent wells, called "céanes," that usually lack cemented walls and are used for the watering of market-gardens [28,29].

To assess the heterogeneity of malaria transmission risk in Dakar, a clear understanding of current ecological requirements for the persistence of productive breeding habitats is necessary.

## Methods

### Study site

Dakar (14°40'20" North, 17°25'22" West), the capital city of Senegal, is located in the Cap-Vert peninsula at the westernmost point of Africa. The estimated population was 1,030,594 inhabitants in 2005, amounting to about 20% of the country's population. The population density is 12,233 inhabitants per km^2^. The altitude peaks at 104 m above sea level (Mamelles). The study was conducted in ten different areas of downtown Dakar and Pikine, one of its satellite city.

**Figure 1:**
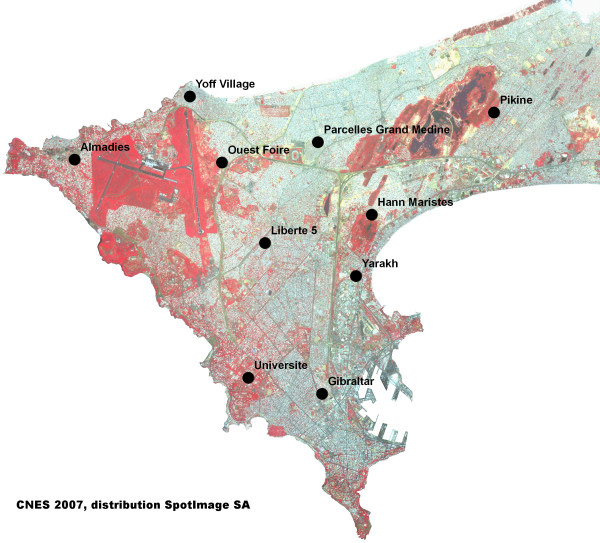
**Cap-Vert peninsula and localization of the ten study areas**.

Site selection was done on the basis of a SPOT-5 (*Satellite Pour l'Observation de la Terre*) satellite image (CNES 2006, Distribution Spot Image SA) acquired in October 2006 (Figure 1) and classified using a supervised technique which allowed to affect each pixel of the image to a land cover. Result of this process provided a map of vegetation, water, bare soils and different types of urban areas. Based on this land cover map, the study areas were sampled in order to cover as many different environments as possible, in terms of type of urbanization and presence of vegetation. Each site was delimited on the ground to cover an area of about 200 × 200 m, depending on the technical and logistical limitations presented by the landscape (Figure 1). Geographic coordinates are given for the centre of each study area.

One study site was located in Pikine (14°45'30"N, 17°23'56"W), an underprivileged satellite city of Dakar. About half of the area is covered with marshland (locally called "niaye"), vegetation and market-gardens whose wells (locally called "céanes") are not reinforced with cement. In the remainder of this area, buildings are individual or collective, structured around a network of unpaved sand roads.

The other study sites were located in the city of Dakar. "Almadies" (14°44'42"N, 17°30'38"W) is located in a privileged residential area. About half of the study area is covered with vegetation and a pond. In the other half, houses are big, air-conditioned and surrounded by large private gardens. The primary road network is paved and the secondary one is unpaved.

"Université" (14°41'22"N, 17°27'49"W) is located on the campus of Dakar Université. Most of the area is covered with low vegetation, trees and a pond. The two existing buildings housing student dormitories are about 70 m long and 4–5 stories high and served by paved asphalt pathways.

"Hann Maristes" (14°43'54"N, 17°25'57"W) is mostly covered by a large park with tall trees and a lake. Outside of the park, the recently-built collective high-rise buildings are spaced out and the road network consists of unpaved sand roads.

"Ouest Foire" (14°44'41"N, 17°28'17"W), close to the airport, is half-covered with low-lying vegetation on sandy ground. On the other half of the site, there are individual houses or small collective buildings, new or under construction. The roads are unpaved. The centre of the area is located in a depression.

"Gibraltar" (14°41'3"N, 17°26'41"W) is in a well-urbanized area, the one closest to the city centre, with medium-size collective buildings and asphalt roads. Vegetation is limited to some trees bordering the main roads.

"Yarakh" (14°42'56"N, 17°26'7"W) was in an area consisting of spontaneous dwellings (huts) built near the railroad and surrounded by an industrial neighbourhood. About half of the area was composed of market-gardens, watered using either "céanes" or cemented wells.

"Liberté 5" (14°43'23"N, 17°27'36"W) is in a well-urbanized residential area, with individual houses of medium size, sometimes with small private gardens. The road network is asphalted. Vegetation is limited to trees bordering main roads and inside the gardens.

Half of the "Parcelles Grand Medine" site (14°44'58"N, 17°26'42"W) is located in a well-planned, urbanized area built on cleaned-up swamps. Individual and collective buildings have two or three storeys and roads are sandy. The other half of the site is in a crowded area, with very narrow sandy pathways. Individual houses are small, often with cemented yards. Nearly no vegetation was found in this part of the study area.

The "Yoff Village" site (14°45'36"N, 17°28'49"W) is a former fisher village, urbanized with small individual and collective buildings of about two storeys, near the seashore. Roads are narrow and sandy. Nearly no vegetation was found in this study area.

### Climate and study period

The Cap-Vert peninsula has a mild sahelian climate. The hot and wet season lasts from June to November, with average temperatures between 24 and 30°C. The cool and dry season lasts from December to May, with average temperatures between 19 and 25°C. The first rains generally occur at the end of June or the beginning of July, and the last ones at the beginning of October. In 2005, 2006 and 2007, the average annual rainfalls were 525, 350 and 248 mm, respectively (data from the Tropical Rainfall Measuring Mission [TRMM] – NASA – ). As a majority of the rain falls in August and September, the study period was chosen to last from September through October 2007, in order to catch the peak of malaria transmission.

### Adult mosquito field sampling

Adult mosquito sampling was carried out once every two weeks during the study period. Human landing catch of adult mosquitoes was conducted both indoors (one catching point) and outdoors (two catching points) in each of the ten study areas, for a total of four nights of capture in each place. Indoor captures were conducted with the window or door slightly ajar. The three catching points were located around the centre of each study area. Within each area, distance between each of the three catching point was about 30 meters. Collectors gave prior informed consent and received yellow fever immunizations and anti-malarial chemoprophylaxis consisting of 100 mg doxycycline per day for the duration the study and one month thereafter. Two collectors were contracted for each catching point to work from 8:00 p.m. to 7:00 a.m., with each one resting every two hours. Collectors were rotated among the catching points on different collection nights to minimize sampling bias.

The mosquitoes were recorded by catching point, date and hour of capture and they were sorted by genera.

### Biting patterns and sporozoite rates

The heads and thoraces of all adult anopheline females caught on human bait were tested by enzyme-linked immunosorbent assay (ELISA) to detect the presence of *P. falciparum *circumsporozoite protein (CSP) [30].

The human biting rate (HBR), also termed aggressiveness, was expressed as the number of female anopheline bites per person per night, averaged for both outdoor and indoor catching points. The CSP index was calculated as the proportion of mosquitoes positive for CSP. Differences between the CSP indices of the various study areas were tested using the Fisher exact test.

The entomological inoculation rate (EIR) was the product of the HBR and the CSP index of mosquitoes collected on humans. Data on seasonal transmission in sahelian climates [31] and previous results [25,26] indicate that *An. gambiae s.l*. biting activity is compatible with malaria transmission occurring only at the end of the rainy season. Consequently, the annual EIR was considered equivalent to the September-October EIR. Thus, annual EIR was calculated as the product of the EIR multiplied by 60 days.

### Adult mosquito species identification

The anopheline mosquitoes were identified morphologically following the Gillies and Coetzee keys [27]. *Culicinae *were identified morphologically following the Edwards keys [32]. All anopheline mosquitoes were stored individually in numbered vials with desiccant and preserved at -20°C in the Medical Entomology Unit of the Institute for Tropical Medicine (IMTSSA), Marseille (France), until processing.

Depending on the number of *Anopheles *caught by site, all specimens or a random sample of a maximum of 100 specimens belonging to the *An*. *gambiae *complex were selected from each study site for identification to species by polymerase chain reaction (PCR) [33]. All CSP-positive anopheline mosquitoes were also tested. Differences in the distribution of species between the study areas were tested using the Fisher exact test.

### Field larval sampling

Each yard within the study areas was searched for open water collection sites. The study areas were visited every week during the eight weeks of the study period, except for the "Université", "Hann Maristes" and "Gibraltar" areas, which were only monitored during the last six weeks of the study period. All the water collections in the ten study areas were examined for larvae. Larvae and pupae were sampled using a standard dipping method [34]. When anopheline specimens were found, larval density was calculated as the number of larvae (all instars) and pupae (further emerged and identified at the laboratory) per dip and recorded for each water collection site. Temporal synchronism in larval density was examined within each area to assess whether the peaks of larval density were synchronized across the breeding sites within each study area. The presence of *Culicinae *larvae was also recorded.

### Larval mosquito data analysis

A random sample of the larvae and all the pupae were taken to the laboratory for growth and emergence. The neonate *Anopheles *were identified morphologically following the Gillies and Coetzee keys [27], stored by date and breeding site in numbered vials with desiccant and preserved at -20°C at the Medical Entomology Unit of the Institute for Tropical Medicine (IMTSSA), Marseille (France), until processing.

All anopheline larvae that were collected in study areas where *An*. *melas *adults had been caught on human bait and that emerged in the laboratory were identified by species following the same PCR protocol.

### Characterization of open water collection sites

Physical, biological and chemical characteristics of the open water collection sites were recorded by the same person, in order to maintain consistency in visual classifications.

Habitat type of all bodies of water were categorized as ditches or puddles, swamp areas, marshes, ponds or lakes, "céanes," cemented wells or basins, man-made water collection sites, waterproof containers or canals. A water collection site was considered temporary when it was found to be dry at least once during the follow-up or during one field visit undertaken at the end of the 2008 dry season, before the first rains. Otherwise, it was recorded as permanent.

Identification of predators was limited to all types of larvivorous fishes, such as guppies, *Gambusia *or *Tilapia*, which are larval predators. Larvivorous fishes were introduced in Dakar in the 1930s and their presence in market-garden wells is recommended by the National Hygiene Service. Their presence was assessed visually.

The perimeter of each body of water was measured using a centimetre for small pools of water (perimeter <5 metres) and estimated using the number of strides (gauged at one metre each) for large bodies of water (perimeter ≥ 5 metres). The area of each body of water was evaluated by approximating the shape as a square, a rectangle, a circle or an ellipse.

The temperature of the water was measured with a mercury-in-glass thermometer immerged for 60 seconds.

Turbidity was estimated by using a graduated transparent bottle with black letters written on the bottom. The bottle was filled with water from the collection site and turbidity was evaluated by the graduation that the water reached before the letters were no longer visible. Graduations ranged from 0 to 26 cm, starting from the top of the bottle, so that a higher value indicated greater turbidity.

The proportion of the water surface covered by vegetation was estimated visually. The vegetation was not classified further, but included water lettuce, water lentils and grass.

The proportion of the water surface exposed to sunlight was estimated visually by assessing the proportion of the water surface shadowed at midday.

Because of recent reports of *An*. *melas *in Dakar [26], salinity was measured for a sub-sample of the observed water collection sites. Two drops of chloroform were added to water samples, which were then transported to the laboratory (Laboratoire des Moyens Analytiques, IRD Bel Air, Dakar) in a cool box containing ice, for analysis with a conductivity meter (Symphony™ SB70C, VWR International^®^) within hours after their collection.

### Statistical analysis

The statistical analyses of the larval collections aimed to identify: 1) the determinants of the presence/absence of anopheline larvae in water collection sites, and 2) the factors associated with the density of anopheline larvae in the breeding sites.

Continuous independent variables were dichotomized at the median. The statistical unit was the weekly measurement of variables for a given water collection site.

In longitudinal studies, some correlation could exist between observations made on the same water collection site. To take into account this interdependence of observations, GEE population-averaged models were used. The within-group correlation structure was chosen as autoregressive of order 1, corresponding to the one-week delay between two observations of the same site.

Similarities could exist between water collection sites in the same study area. Thus, a dummy variable corresponding to the study sites was forced in all univariate and multivariate analyses to take into account the fact that several water collection sites belonged to the same study area.

The presence/absence of larvae in the water collection sites was analysed using a logistic regression model. The larval density by breeding site was analysed using a negative binomial regression model. The dependant variable was the number of larvae per dip minus 1, in order to overcome the exclusion of zero-values and take into account the number of dips sampled at each water collection site.

The variables associated with the presence or the density of larvae with a p-value < 0.25 in univariate analysis were retained for multivariate analysis. A backward stepwise selection procedure was applied in the final model to keep variables with a p-value < 0.05.

Adult densities were estimated at the study site level by the total number of *Anopheles *caught indoors and outdoors during one night of capture. The larval density index at each study site was estimated as follows. The product of the larval density multiplied by the estimated water surface was calculated for each breeding site within one week before the night of mosquito capture. The sum of these products over all the water collection sites was considered as the larval density index for the study area. Correlations between larval density index and adult densities were then examined for each study area. Correlations were also researched between raw larval densities and adult densities and between the estimated water surface and adult densities. All analyses were performed with STATA 9.0 (Stata-Corp LP).

## Results

### Adult mosquito collection

A total of 19,451 mosquitoes (74.18% *Culex quinquefasciatus*, 15.47% *Culex tritaeniorynchus*, 5.66% *An. gambiae s.l*., 4.22% *Aedes aegypti*, 0.05% *Anopheles pharoensis*) were caught during 120 person-nights of collection on human bait. A total of 1,101 *An. gambiae s.l*. were collected (Table 1).

**Table 1:** Distribution by genus and species of adult mosquitoes collected on humans in the ten study areas of Dakar in September-October 2007; there were two outdoor catching points (80 person-nights collection) and one indoor catching point (40 person-nights collection) for each site.

	Outdoors (2 catching points per study site)	Indoors (1 catching point per study site)	% Indoors*	Total	% of total population
*Anopheles gambiae s.l*.	870	231	35%	1101	5.7%
*Anopheles pharoensis*	9	1	18%	10	0.05%
*Culex quinquefasciatus*	9393	5035	52%	14428	74.2%
*Culex tritaeniorhynchus*	2606	404	24%	3010	15.5%
*Aedes aegypti*	770	51	12%	821	4.2%
*Aedes metallicus*	1	0	0%	1	0.01%
*Mansonia sp*	62	18	37%	80	0.4%

Total	13711	5740	42%	19451	100%

* Percentages of indoor bites were calculated using the number of outdoor bites averaged for one catching point.

### Biting behaviour of *An. gambiae s.l*

**Figure 2:**
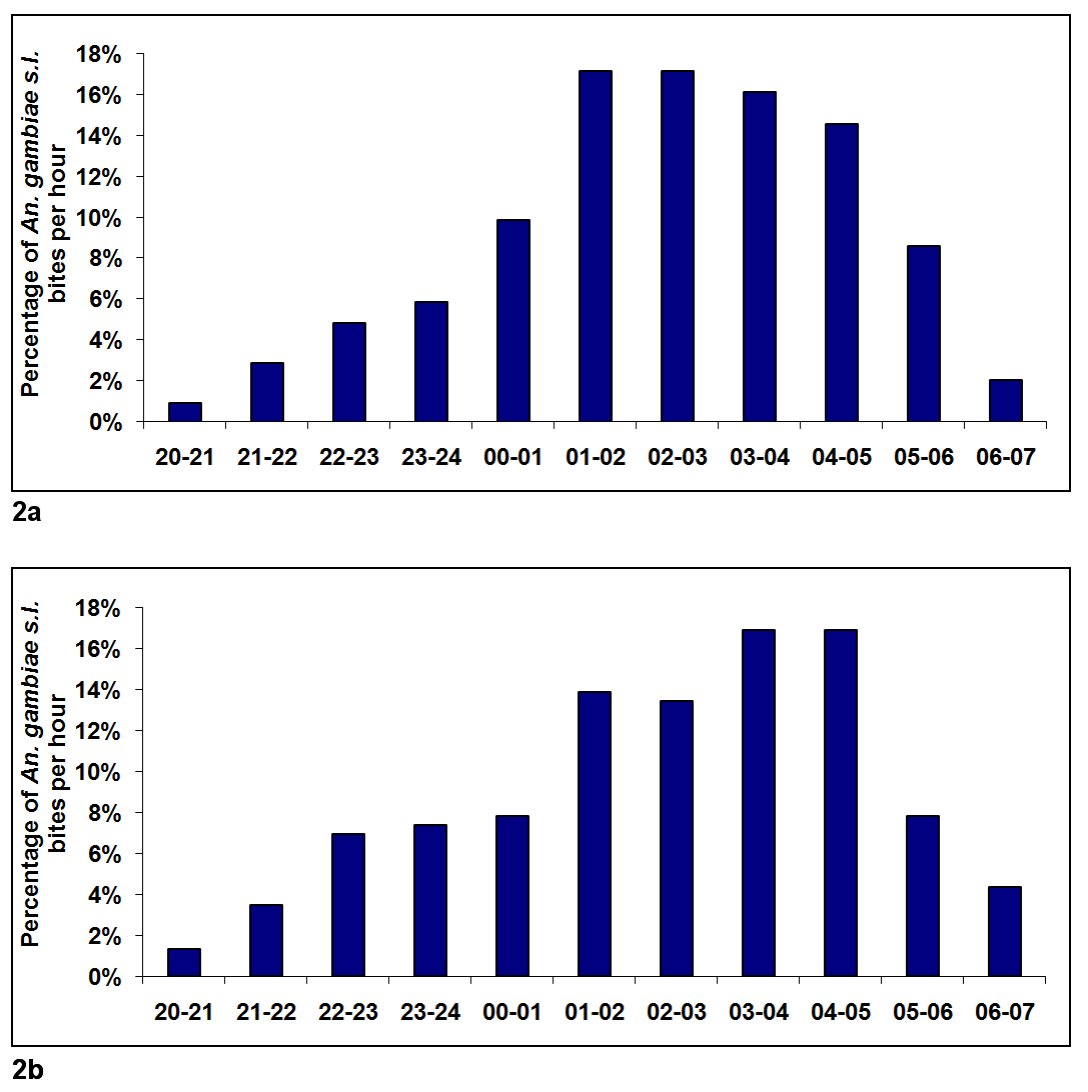
**Hourly distribution of *An. gambiae s.l*. bites outdoors (Figure 2a) and indoors (Figure 2b) in the ten study areas of Dakar in September-October 2007**.

The total number of *An. gambiae s.l*. caught during the 12 person-nights of collection in each of the study areas ranged from one in Yoff Village to 524 in Almadies (Table 2). Among the 1,101 *An. gambiae s.l*. caught on human collectors during the eight weeks of follow-up in the 10 study areas, 870 were caught outdoors (two catching points for each night of capture) and 231 were caught indoors (one catching point for each night of capture) (Table 2). Using the number of outdoor bites averaged for one catching point only, it has been found that 35% of all bites were received indoors. Considering all ten study areas together, the peak biting time was between 1:00 a.m. and 5:00 a.m. both outdoors (Figure 2a) and indoors (Figure 2b). *Anopheles gambiae s.l*. caught during this time range accounted for 65% and 61% of the total number caught outdoors and indoors, respectively, during the whole night.

**Table 2:** Distribution of adult *An. gambiae s.l*. collected on humans in two outdoor catching points (8 person-nights collection per study site) and one indoor catching point (4 person-nights collection per study site) in the ten study areas of Dakar in September-October 2007.

Study sites	Outdoors (2 catching points per study site)	Indoors (1 catching point per study site)	Total
Almadies	478	46	524
Pikine	150	78	228
Université	111	27	138
Hann Maristes	35	29	64
Ouest Foire	32	17	49
Gibraltar	24	19	43
Yarakh	31	11	42
Liberté 5	6	2	8
Parcelles Grand Medine	2	2	4
Yoff Village	1	0	1

Total	870	231	1101

### Molecular identification of *An. gambiae s.l*. caught on human bait

Depending on the total number of specimens collected in each study area, the random sample selected for molecular identification of species represented 19% to 100% of the total adult *An. gambiae s.l*. caught. Among the 496 specimen tested by PCR, the *An*. *gambiae *complex population was composed of *An*. *arabiensis *(94.8%) and *An*. *melas *(5.2%). The detailed percentages of *An*. *arabiensis *and *An*. *melas *per study area are presented in Table 3. Differences among areas were significant (exact Fisher test; p < 0.001).

**Table 3:** Proportions of *An. arabiensis *and *An. melas *among the *An. gambiae s.l*. collected on humans in the ten study areas of Dakar in September-October 2007.

	Number of *An. gambiae s.l*. processed by PCR	Number of *An. arabiensis*	Proportion of *An. arabiensis*	Number of *An. melas*	Proportion of *An. melas*
Almadies	102	102	100%	0	0%
Pikine	99	78	79%	21	21%
Université	93	93	100%	0	0%
Hann Maristes	62	58	94%	4	6%
Ouest Foire	44	44	100%	0	0%
Gibraltar	43	43	100%	0	0%
Yarakh	41	41	100%	0	0%
Liberté 5	7	7	100%	0	0%
Parcelles Grand Medine	4	3	75%	1	25%
Yoff Village	1	1	100%	0	0%

Total	496	470	95%	26	5%

### HBR, CSP and EIR

**Figure 3:**
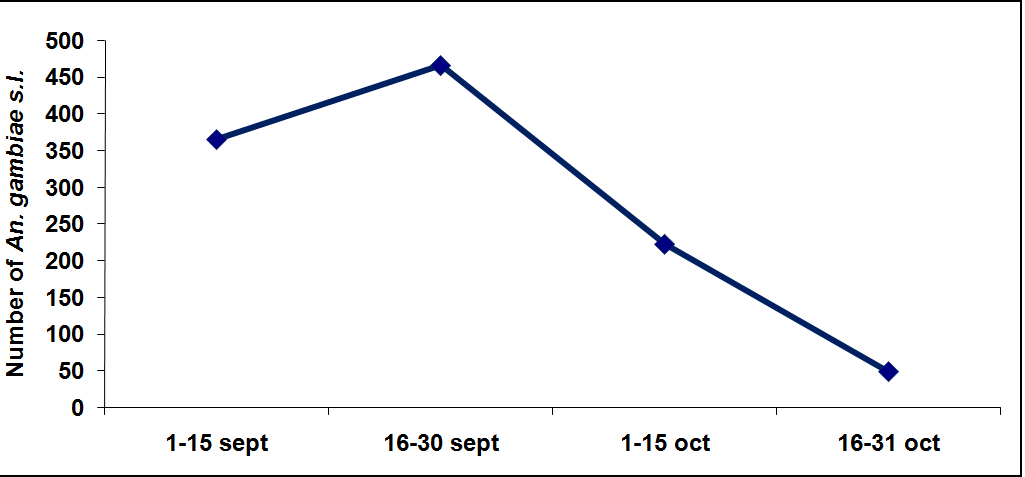
**Temporal distribution of adult *An. gambiae s.l*. collected in two catching points outdoors and one catching point indoors in the ten study areas of Dakar in September-October 2007**.

HBR was calculated as the number of *Anopheles *bites received per person per night, taking into account figures for both indoor and outdoor bites, averaged over the eight weeks of follow-up. *An. gambiae s.l*. HBR ranged from 0.1 bites per person per night in Yoff Village to 43.7 in Almadies. The highest recorded HBR was 211 bites per person per night, outdoors at the end of September in Almadies. The highest aggressiveness was recorded in the second half of September (Figure 3), when 42% of all adult *An. gambiae s.l*. were caught.

All of the 1,101 *An. gambiae s.l*. were processed by ELISA for *P. falciparum *antigen detection and seven were found to be positive. None of the *An. melas *were found to be infected with *P. falciparum*. The infected *An. arabiensis*. were caught outdoors in Almadies, Pikine, Ouest Foire and Yarakh, in September or in the first fortnight of October. The mean CSP index was 0.64% (95% CI = 0.19% – 0.96%). No significant differences were found in CSP indices between the study areas (exact Fisher test; p = 0.790). Thus, EIR, annual EIR and the calculated period (in days) between two *An. arabiensis *infective bites were calculated using the mean CSP index. Annual EIR ranged from 0 infective bites in Yoff Village to 16.8 in Almadies. HBR and EIR figures for *An. arabiensis *are presented in Table 4. Differences between *An. arabiensis *annual EIR and *An. gambiae s.l*. annual EIR were found mainly for Pikine were it decreased form 7.3 to 5.8 taking *An. arabiensis *HBR only.

**Table 4:** HBR, EIR, annual EIR and calculated period in days (1/EIR) between two infected bites by *An. Arabiensis *in the ten study areas of Dakar in September-October 2007 (mean CSP index = 0.64%).

Zone	HBR	EIR	Annual EIR	Calculated period (in days) between 2 infected bites
Almadies *	43.7	0.28	16.8	4
Pikine *	15.0	0.10	5.8	10
Université	11.5	0.07	4.4	14
Hann Maristes	5.0	0.03	1.9	31
Ouest Foire *	4.1	0.03	1.6	38
Gibraltar	3.6	0.02	1.4	43
Yarakh *	3.5	0.02	1.3	45
Liberté 5	0.7	0.00	0.3	223
Parcelles Grand Medine	0.2	0.00	0.1	694
Yoff Village	0.1	0.00	0.0	1563

* Study areas in which infected *Anopheles *have been caught.

### Larval sampling

A total of 54 open bodies of water were monitored weekly, during four to eight weeks. Several types of open water collection sites were found: 23 ditches or puddles, seven swamp areas, marshes, ponds or lakes, 16 "céanes," cemented wells or basins, three man-made water collection sites, four waterproof containers and one canal.

Of the water collection sites, 34 (63%) were temporary (23 ditches or puddles, four swamp areas, marshes, ponds or lakes, three man-made water collection sites and four waterproof containers) and 20 (37%) were permanent (one canal, three swamp areas, marshes, ponds or lakes, 16 "céanes," cemented wells or basins). Most (79%) of 34 temporary collection sites and many (40%) out of 20 permanent collection sites were observed to be habitats for anopheline larvae at least once during follow-up.

More than half (33, 61.1%) of the bodies of water were found to be breeding sites for anophelines on at least one observation during the follow-up period but only six (11.1%) harboured larvae for the whole duration of the follow-up. No breeding habitats were found in Liberté 5 and Yoff Village. In Parcelles Grand Medine, one breeding site was found outside of the 200 × 200 m area. In the other study areas, the number of water collection sites ranged from four to eight. Most of the positive collection sites for mosquitoes were located in Almadies, where every body of water was observed to be a breeding site at least once during follow-up. In breeding habitats, the density of larvae and pupae ranged from 0.05 to 35 per dip (mean = 6.05, 95% CI = 4.85 – 7.25).

No temporal synchronism was observed in the larval density of breeding sites within each area and the peaks in larval density were not synchronized within each study area.

### Identification of *An. gambiae s.l*. reared from larval samples

Identification of species by PCR amplification showed that 388 adult *An. gambiae s.l*. specimens reared from larval samples were *An*. *arabiensis*. No *An*. *melas *mosquitoes were identified.

### Characterization of open water collections

A total of 389 observations of water collection sites were recorded, among which 130 (33.4%) were positive for *Anopheles *in immature stages (*i.e*,. larvae or pupae), 196 (50.4%) were negative and 63 (16.2%) corresponded to a water collection site that had dried up. The percentage of observations in which the water collection site had dried up increased from 0 to 31.5% over the course of follow-up. Only the 326 observations of sites containing water (and not the observations of dried-up sites) were taken into account in the following analysis.

Larvivorous fishes were found in 180 (62%) observations of 42 water collection sites that were negative for larvae and pupae and 112 (48%) observations of 36 water collection sites that were positive. Description of the quantitative physical, biological and chemical parameters recorded for the open bodies of water are presented in Table 5. Based on 99 observations, mean salinity was 1.34 g/l (95% CI = 1.07 – 1.6]) and ranged from 0 to 6.8 g/l.

**Table 5:** Description of the quantitative physical, biological and chemical parameters recorded for the open water collection sites in the ten study areas of Dakar, depending on breeding status.

Parameters		Anopheline larvae and pupae absent	Anopheline larvae and pupae present
Perimeter (metres)	n collection sites = 54	43	36
	n observations = 326	196	130
	Range	0.3 – 579.3	0.4 – 1973.6
	Mean and 95% CI	36.3 [26.0–46.7]	148.4 [76.6–220.2]
	25–50–75 percentiles	6.3 – 16.7 – 39.5	9.8 – 29.7 – 75.6
			
Temperature (°C)	n collection sites = 54	40	34
	n observations = 248	152	96
	Range	25.0 – 39.0	25 – 40
	Mean and 95% CI	29.5 [29.1–29.9]	31.7 [30.9–32.5]
	25–50–75 percentiles	28 – 29 – 30	28 – 31 – 34
			
Turbidity	n collection sites = 54	35	31
	n observations = 225	135	90
	Range	0 – 26	0 – 26
	Mean and 95% CI	9 [7-10]	16 [14-17]
	25–50–75 percentiles	0 – 4 – 17	8 – 19 – 23
			
Surface vegetation (%)	n collection sites = 54	43	36
	n observations = 324	194	130
	Range	0 – 100	0 – 90
	Mean and 95% CI	32 [27-36]	18 [13-22]
	25–50–75 percentiles	2 – 10 – 60	0 – 5 – 20
			
Sunlight (%)	n collection sites = 54	42	34
	n observations = 319	193	126
	Range	0 – 100	0 – 100
	Mean and 95% CI	63 [59–67]	69 [64–74]
	25–50–75 percentiles	50 – 70 – 95	50 – 70 – 100

### Determinants of the presence/absence of larvae and larval density

Three water collection sites were observed only once because they dried up after the first week of observation, although all three harboured larvae when they were observed. The 19 observations corresponding to these locations were excluded from the statistical analysis, as the fit of GEE models with an autoregressive correlation matrix required at least two observations of the same water collection site.

Thus, 323 observations with known breeding status and 123 observations of breeding sites with known larval density were considered for the following analysis.

Tables 6 and 7 provide the results of univariate analyses for the presence/absence of *Anopheles *larvae and larval density, respectively. The total number of observations may differ from the one in Table 5 because of the restriction introduced by the fit of the GEE statistical model. Analyses for the presence/absence of *Anopheles *larvae were completed based on observations for which the larval status was known, even if the larval density was unrecorded. Thus, the total number of observations may differ between Tables 6 and 7.

**Table 6:** Factors related to the presence/absence of anopheline larvae.

Habitat characteristics	Number of obs.	Number of obs. positive for anopheline larvae	% obs. anopheles positive	OR	95% CI	p-value
**Zone**						**< 0.001**
Yarakh	92	3	3%	1		
Parcelles Grand Medine *	7	1	14%	2.51	0.06 – 97.52	**0.623**
Gibraltar	4	1	25%	7.71	0.34 – 173.14	**0.198**
Pikine	91	29	32%	11.83	2.31 – 60.62	**0.003**
Ouest Foire	36	23	64%	41.99	6.96 – 253.38	**<0.001**
Université	21	15	71%	65.20	8.70 – 488.74	**<0.001**
Hann Maristes	23	18	78%	68.69	9.11 – 517.73	**<0.001**
Almadies	49	38	78%	95.02	15.33 – 589.07	**<0.001**
Liberté 5	0	0	0%	-		
Yoff Village	0	0	0%	-		

						

**Habitat type**						**<0.001**
"Céanes", cemented weels or basins	128	5	4%	1		
Man-made water collections	18	5	28%	2.45	0.29 – 20.83	0.412
Canals	8	4	50%	3.02	0.21 – 43.62	0.417
Waterproof containers	15	6	40%	4.43	0.48 – 40.62	0.188
Swamp areas, marshes, ponds or lakes	44	24	55%	7.82	1.40 – 43.73	0.019
Ditches or puddles	110	84	76%	24.82	4.63 – 130.82	<0.001

						

**Period**						**0.793**
Weeks 1 – 4	162	62	38%	1		
Weeks 5 – 8	161	66	41%	1.09	0.58 – 2.03	

**Temporary collection**						**0.058**
No	155	22	14%	1		
Yes	168	106	63%	2.67	0.97 – 7.37	

**Perimeter (metres)**						**0.168**
<20 m	157	54	34%	1		
>= 20 m	166	74	45%	0.59	0.27 – 1.25	

**Water temperature (n = 243)**						**0.077**
<30°C	120	31	26%	1		
>= 30°C	123	62	50%	1.96	0.93 – 4.13	

**Turbidity (n = 222)**						**0.808**
<13 (clear)	106	26	25%	1		
>= 13 (turbid)	116	62	53%	1.09	0.54 – 2.21	

**Surface vegetation (%, n = 320)**						**0.078**
<20%	195	93	48%	1		
>= 20%	125	35	28%	0.44	0.18 – 1.10	

**Sunlight (%, n = 317)**						**0.974**
<80%	202	73	36%	1		
>= 80%	115	52	45%	0.99	0.40 – 2.45	

**Presence of predator (n = 287)**						**0.641**
No	132	55	42%	1		
Yes	155	54	35%	0.84	0.41 – 1.74	

**Presence of *Culicinae *larvae (n = 293)**						**<0.001**
No	211	48	23%	1		
Yes	84	58	69%	5.46	2.55 – 11.66	

Adjusted (by study area) odds ratio (OR) estimated by GEE logistic regression model. Bivariate analysis: 323 observations of water collection sites unless otherwise indicated.

* Water collections found outside of the 200 × 200 m study area

**Table 7:** Factors related to anopheline larval density.

Habitat characteristics	Number of obs.	Number of dips	Total number of anopheline larvae	Anopheline larval density (per dip)	RR	95%CI	p-value
**Zone**							**0.028**
Hann Maristes	18	209	208	1.00	ref.		
Pikine	27	140	357	2.55	1.66	0.77 – 3.60	**0.198**
Université	14	54	246	4.56	2.23	0.91 – 5.46	**0.080**
Yarakh	2	18	110	6.11	2.28	0.41 – 12.76	**0.348**
Ouest Foire	22	93	578	6.22	3.09	1.38 – 6.95	**0.006**
Almadies	38	179	1309	7.31	4.09	1.98 – 8.45	**<0.001**
Gibraltar	2	9	160	17.78	8.10	1.48 – 44.39	**0.016**
Parcelles Grand Medine *	0	-	-	-			
Liberté 5	0	-	-	-			
Yoff Village	0	-	-	-			

							

**Habitat type**							**<0.001**
"Céanes", cemented weels or basins	8	53	126	2.38	ref.		
Waterproof containers	5	17	30	1.76	2.56	0.45 – 14.72	0.291
Swamp areas, marshes, ponds or lakes	22	116	467	4.03	14.76	3.76 – 57.88	<0.001
Ditches or puddles	80	488	2137	4.38	15.96	4.44 – 57.36	<0.001
Man-made water collections	4	16	82	5.13	17.45	2.62 – 116.14	0.003
Canals	4	12	126	10.50	22.50	3.90 – 129.71	<0.001

							

**Period**							**0.762**
Weeks 1 – 4	57	328	1775	5.41	ref.		
Weeks 5 – 8	66	374	1193	3.19	0.94	0.63 – 1.40	

**Temporary collection**							**0.292**
No	23	134	379	2.83	ref.		
Yes	100	568	2589	4.56	1.44	0.73 – 2.85	

**Perimeter (metres)**							**0.755**
<20 m	49	226	1078	4.77	ref.		
>= 20 m	74	476	1890	3.97	0.93	0.59 – 1.46	

**Water temperature (n = 93)**							**<0.001**
<30°C	33	228	475	2.08	ref.		
>= 30°C	60	307	1611	5.25	3.34	1.97 – 5.68	

**Turbidity (n = 88)**							**0.592**
<13 (clear)	28	142	700	4.93	ref.		
>= 13 (turbid)	60	392	1261	3.22	0.87	0.52 – 1.46	

**Surface vegetation (%)**							**0.001**
<20%	89	504	2596	5.15	ref.		
>= 20%	34	198	372	1.88	0.41	0.24 – 0.70	

**Sunlight (%, n = 120)**							**0.674**
<80%	72	447	1194	2.67	ref.		
>= 80%	48	244	1665	6.82	1.13	0.64 – 2.00	

**Presence of predator(n = 104)**							**0.865**
No	48	308	1326	4.31	ref.		
Yes	56	286	1245	4.35	0.96	0.59 – 1.56	

**Presence of *Culicinae *larvae (n = 106)**							**0.016**
No	51	291	1259	4.33	ref.		
Yes	55	321	1033	3.22	1.71	1.10 – 2.64	

Adjusted (by study area) risk ratio (RR) estimated by GEE negative binomial regression model. Bivariate analysis; 123 observations of anopheline breeding sites, unless otherwise indicated.

* Water collections found outside of the 200 × 200 m study area

Table 8 provides the results of the multivariate analyses for the presence/absence of *Anopheles *larvae and for larval densities. Salinity was not included in the multivariate analysis because of the small number of observations for which this parameter was recorded.

**Table 8:** Factors related to the presence/absence of larvae and larval densities, adjusted by study area.

**Presence/absence of anopheline larvae (n = 293)**, GEE logistic regression model			
Habitat characteristics	Odds Ratio	95% CI	p-value

			<0.001
**Study areas**			
Parcelles Grand Medine	1		
Pikine	1.96	0.08 – 49.21	0.682
Yarakh	2.10	0.05 – 93.07	0.702
Hann Maristes	9.19	0.35 – 238.37	0.182
Ouest Foire	13.88	0.51 – 378.05	0.119
Almadies	35.56	1.47 – 862.54	0.028
Université	46.69	1.50 – 1449.39	0.028
**Habitat type**			
Man-made water collections	1		
Waterproof containers	1.51	0.09 – 24.72	0.771
Canals	1.53	0.06 – 41.92	0.801
"Céanes", cemented weels or basins	3.65	0.23 – 58.63	0.360
Swamp areas, marshes, ponds or lakes	10.14	1.14 – 90.28	0.038
Ditches or puddles	33.47	4.05 – 276.48	0.001
**Presence of Culicinae larvae**			<0.001
No	1		
Yes	8.24	3.26 – 20.86	

**Larval density (n = 86)**, GEE binomial regression model			

Habitat characteristics	Risk Ratio	95% CI	p-value

			<0.001
**Study areas**			
Hann Maristes	ref.		
Université	1.15	0.29 – 4.54	0.838
Pikine	2.00	0.86 – 4.66	0.107
Ouest Foire	5.03	2.09 – 12.53	0.001
Almadies	5.48	2.37 – 12.68	<0.001
Yarakh	10.98	1.46 – 82.75	0.020
**Habitat type**			
Waterproof containers	ref.		
Canals	3.78	0.61 – 23.53	0.155
"Céanes", cemented weels or basins	9.53	1.08 – 83.91	0.042
Ditches or puddles	10.82	2.83 – 41.41	0.001
Swamp areas, marshes, ponds or lakes	23.29	5.16 – 105.04	<0.001
Man-made water collections	48.16	4.55 – 509.71	0.001
**Water temperature**			<0.001
<30°C	ref.		
>= 30°C	3.13	1.67 – 5.84	
**Surface vegetation (%)**			0.007
<20%	ref.		
>= 20%	0.36	0.17 – 0.75	
**Presence of *Culicinae *larvae**			0.019
No	ref.		
Yes	2.29	1.14 – 4.60	

Multivariate analysis with GEE logistic regression model or negative binomial regression model.

Temporary nature, habitat type, perimeter, water temperature, percentage of surface vegetation and co-occurrence of *Culicinae *larvae were significantly associated with the presence/absence of larvae in bivariate analysis (taking into account the study area effect). The only variables remaining in the model after applying the backward stepwise selection were the presence of *Culicinae *larvae, the habitat type and the study area.

Larval density was significantly associated with habitat type, water temperature, percentage of surface vegetation and co-occurrence of *Culicinae *larvae in bivariate analysis (taking into account the study area effect). These four parameters remained significant in the multivariate analysis.

### Correlation between larval and adult densities

**Figure 4:**
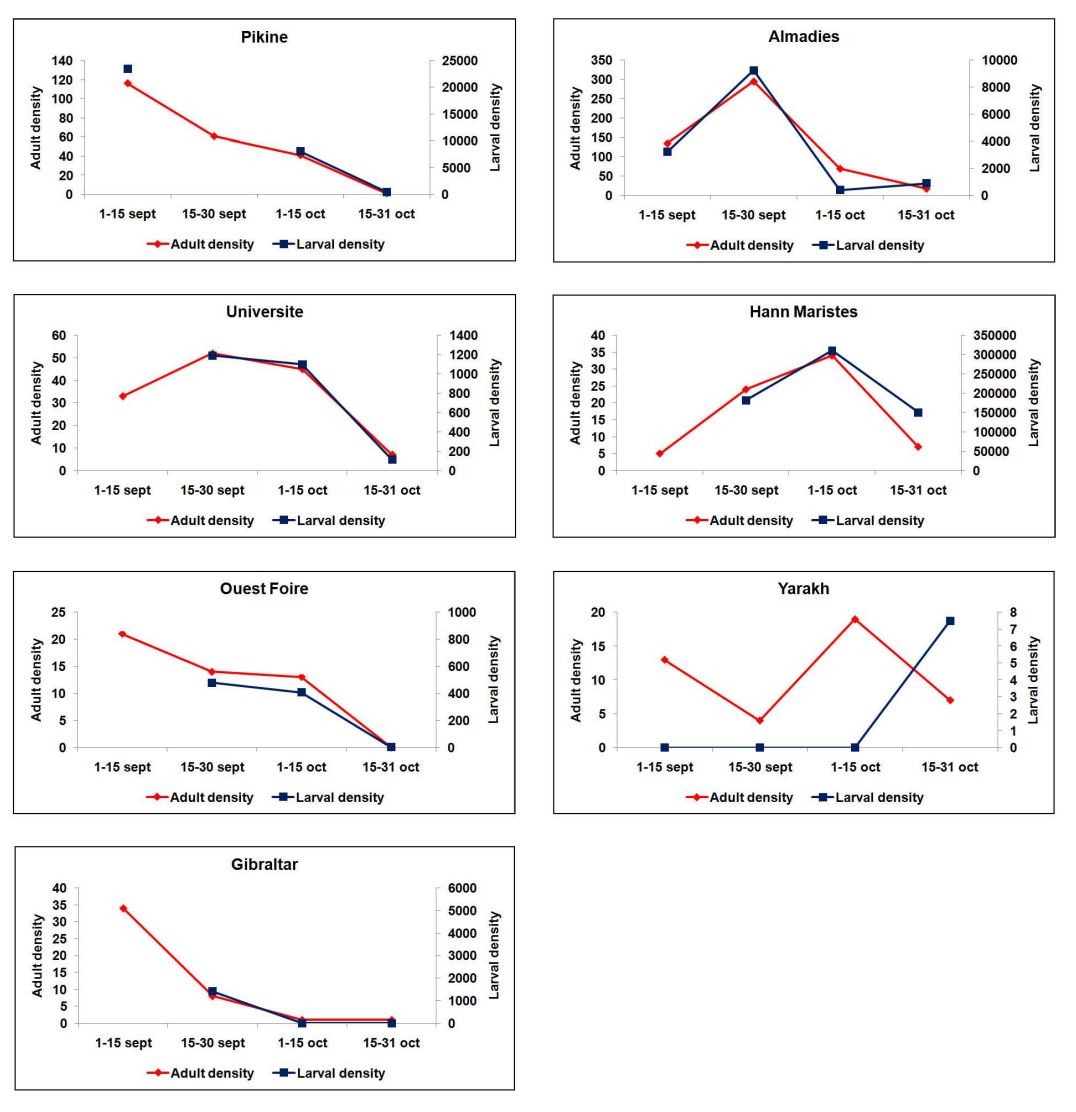
**Graphical representation of *An. gambiae s.l*. aggressiveness (left scale, total number of Anopheles caught indoors and outdoors) and larval densities observed one week before adult catch and ponderated by the breeding habitats surface (right scale, larval density × surface water for all the breeding sites), for seven out of ten study areas of Dakar in September-October 2007**.

Figure 4 shows the temporal variations in adult density and larval density index the week before the adult captures, in the study districts for which the larval and adult densities were high enough for comparison. Liberté 5, Parcelles Grand Medine and Yoff Village are not represented, as no breeding sites were found within the 200 × 200 m study area. In Université, Hann Maristes and Gibraltar, only three values are available for larval density because the follow-up lasted six weeks. In Pikine and Ouest Foire, one value for larval density was missing.

The graphical examination highlights a very close parallel between larval and adult densities in six out of seven study areas. Graphical comparisons of the temporal variations of the adult density with the raw larval density on one hand and the surfaces of the water collections on the other hand did not show this close parallel.

## Discussion

### Heterogeneity in local malaria transmission

The results provide evidence of malaria transmission in downtown Dakar and its nearby suburb. The rate of infection of the *An. gambiae s.l*. caught on human bait at the end of the 2007 rainy season was 0.64%.

Spatial heterogeneity of human biting rates was very marked, with HBR up to 400 times higher in one area than in other areas located a few kilometres away. HBR ranged from 0.1 bites per person per night in Yoff Village to 43.7 in Almadies. Heterogeneity of the CSP index could not be demonstrated, as no significant differences were found in the rates of infection among the study areas. Nevertheless, the high heterogeneity in anopheline aggressiveness led to a high heterogeneity in the risk of malaria transmission between inhabitants of different study areas. Annual EIR could be calculated using the figures for September and October, as the majority of *Anopheles *bites are received during this period of the year [25,26]. EIR ranged from 0 infective bites per person per year in Yoff Village to 16.8 in Almadies (one infective bite every four days during the transmission season). Thus, in Dakar, no mean figures for transmission would provide a comprehensive picture of the situation; risk evaluations should be conducted on a local scale.

The highest HBR (43.7) and the highest annual EIR (16.8) of the present study were recorded in Almadies. In one of the outdoor catching points, the HBR reached 211 bites per person in one night at the end of September. This high HBR could be explained by the large number of productive breeding sites found in this study area, and the high density of larvae in these habitats. The large amount of vegetation in this area could also favour the survival of adult mosquitoes. Furthermore, the population density in the area was low and the type of housing in this privileged residential area meant that most of the population slept in air-conditioned rooms, which could lead to a concentration of bites on the few night watchmen sleeping outdoors.

Annual *An. arabiensis *EIRs in Pikine, Université, Hann Maristes and Ouest Foire ranged from 1.6 to 5.8 and were consistent with the results of the meta-analysis by Robert *et al *[3], which showed that mean annual EIR was 7.1 in sub-Saharan African city centres and that more than two-thirds of the studies reported an EIR <4 infective bites per year. In those areas, figures were also consistent with measurements conducted in two areas of Dakar in 2005–2006, which found EIRs of 3 to 9.5 infective bites per person per year [26]. Breeding habitats were found in these four areas and the large amount of vegetation may have favoured adult mosquito survival.

In Gibraltar, the annual EIR was 1.4 but none of the small water collection sites of the area provided a breeding habitat for more than one week during follow-up. Moreover, Gibraltar is a well-urbanized area with very little vegetation, which is probably not favourable for adult mosquito longevity. Extended investigations are needed to detect the breeding sites of the adult *Anopheles *caught on human bait in this area.

The annual EIR was 1.3 in Yarakh, but few breeding sites were located within the study area. The lack of a parallel between adult aggressiveness and the larval densities in this setting also suggests that the adult mosquitoes caught in this study area had their breeding site in the neighbourhood. The large amount of vegetation in the area also probably favoured the survival of adult mosquitoes.

In Liberté 5, Parcelles Grand Medine and Yoff Village, annual EIRs were below one infective bite per person per year. No infected specimens where caught in these areas, but even when applying the mean CSP index, the EIR was not compatible with transmission due to the low aggressiveness. Although no breeding sites were detected in Liberté 5, HBR was not nil. This could be explained by the presence of trees bordering the main roads and inside the gardens, which could constitute resting sites for adult *Anopheles *coming from sources located outside the study perimeter.

In Parcelles Grand Medine and Yoff Village, the EIR was close to zero. No breeding sites were detected in the 200 × 200 m area and vegetation was very sparse, consistent with the very small number of specimens caught on human bait.

In an ecological point of view, areas where the presence of vegetation was important showed the highest vector densities (ex: Almadies, Pikine), favouring the presence of larval habitats and probably of resting places. On the contrary, areas where percentage of urbanization was high showed a lower number of adult *Anopheles*. Type of soil also had an importance as sand and asphalt (ex: Parcelles Grand Medine and Yoff Village) did not favour persistence of water, compared to mud and swamp areas (ex: Almadies and Yoff).

Among the ten study areas, four (Université, Liberté 5, Gibraltar, Yarakh) were located close to the areas studied by Diallo *et al *in 1994–95 and 1996–97 [17,25]. The HBRs measured in the present study were higher than those recorded during those past studies. This difference must be interpreted with caution, as the sites of mosquito captures were not exactly identical. Furthermore, the peak of *An. gambiae s.l*. aggressiveness lasts for little longer than one month, with a monthly frequency of capture, the previous studies could have missed this peak. The previously published results did not show any infected *Anopheles*, whereas the present study shows that transmission exists.

Transmission was demonstrated in Pikine 30 years ago [14,24] and the present study provides new evidence of local transmission (annual EIR = 5.8) at an intermediate level compared to the results published in 1979–80 (annual EIR = 43) and 1987–88 (annual EIR = 0.38).

Malaria transmission has not been recently studied in the other study areas of the project, so the present study provides new entomological data for Dakar's districts.

### Endo/exophagic behaviour

Important differences existed between the study areas in terms of the endo/exophagic behaviour of *An. gambiae s.l*. but results should be interpreted carefully. Large differences were recorded between outdoor catching sites in the same study area during the same night. As logistical constraints limited the number of indoor catching points to one per site, the experimental design probably did not allow for results representative of adult *An. gambiae s.l*. behaviour in each study area. Using more outdoor and indoor catching points would allow for representative estimates of HBRs in each study site.

### Anopheline species

Among the total female mosquitoes caught on human bait, 5.71% were *Anopheles *(5.66% *An. gambiae s.l*., and 0.05% *An. pharoensis*). This percentage was higher than that recorded in 1994–95 in the south district of Dakar, where the percentage of *Anopheles *was 0.7% [25], and in 1996–97 in the central district, where this proportion was 1.5% [17]. In Pikine in 1987–88, this percentage rose to more than 20% when collections were done on human bait [14].

In the present study, *An*. *arabiensis *and *An*. *melas *were the only representatives of the *An*. *gambiae *complex caught on human bait. No *An. gambiae s.s*. was found, as had been the case in Dakar in 2005–2006 [26]. *Anopheles arabiensis *was the main species identified during September and October 2007 and it accounted for 94.2% of the *An*. *gambiae s.l*. caught. *Plasmodium falciparum *infection was detected only in *An*. *arabiensis *specimens. The predominance of *An. arabiensis *was consistent with previous results from the Cap-Vert peninsula [14,24,25,35].

In 1979–80, in Pikine, one *An. melas *was caught out of 92 *An. gambiae s.l*. adult females [24]. *Anopheles melas *was then captured for the first time in downtown Dakar in 2005–2006 and accounted for 2% of the *An. gambiae s.l*. population [26]. In the present study, the percentage of *An. melas *was 5.2% on average, and the peak was greater than 20% in Pikine. As it is known that *An. melas *is not as good a vector as *An. arabiensis *[36], the relationship between aggressiveness and related malaria transmission risk must be interpreted carefully. This relationship will depend on the proportion of *An. melas *and also on the geographic areas. In the present study area of Pikine, the annual EIR calculated on *An. gambiae s.l*. aggressiveness was 7.3 infective bites, but the EIR calculated on *An. arabiensis *aggressiveness was only 5.8 infective bites.

In the breeding habitats, classification down to the species level of the sampled anopheline larvae identified only *An. arabiensis*. The places where *An. melas *were caught were those closest to the large marshy area locally called "niaye." In this area close to the Atlantic Ocean, the water table is high. In the eighties, Vercruysse *et al *[24] noted that this water table in Pikine was not brackish and that the first salty water collections were observed more than 10 km away from the study's capture points. Thirty years later, the situation could have changed so that the water table now provides habitats for *An. melas *larvae.

However, no *An. melas *larvae were found in the breeding sites we surveyed. There is no report in the literature of *An. melas *larval habitats in Dakar or Pikine. Tolerance of *An. melas *larvae for salinity ranges from 5 to 37 g/l [37]. The maximum salinity recorded in the present study was 6.8 g/l, which would have been favourable for *An. melas *breeding. Further investigations are needed to detect larval habitats that might have been located outside of the present study areas.

*Anopheles pharoensis *accounted for only 0.05% of the total anopheline population and were captured mainly in Hann Maristes. *Anopheles pharoensis *had comprised 4% of the adult anopheline population in Pikine in 1979–80 [24] but only one specimen was caught in the present study in 2007, perhaps indicating a change in the mosquito population. Even though *An. pharoensis *has been suggested as a significant vector in the Senegal River basin [38], it is generally not of epidemiological significance as a malaria vector in Senegal [39]. The low density measured in the present study confirms this fact in the capital.

*Anopheles ziemanni *was previously caught on human bait in Senegal, but in very low numbers [40]. In the present study, no adult specimens were caught on human bait. *Anopheles ziemanni *larvae have already been reported in Pikine [24] and accounted for 14% of the sampled immature stage mosquitoes in the céanes of Dakar [28]. In the present study, no *An. ziemanni *was found among the sampled anopheline larvae. The population of *An. ziemanni *could have changed but the malaria risk would not be affected, as the human blood index of this species'is usually low and it is known only as a secondary or incidental vector [27].

### Larval habitats

Among the 54 water collection sites monitored during the present study, several factors were found to be associated with the occurrence and abundance of anopheline larvae. The presence of larvae and the larval density were strongly associated with the study areas, demonstrating high spatial heterogeneity. This was consistent with the selection of the study areas, which aimed to cover maximal ecological diversity. Thus, adjusting further statistical models for the study area would make it possible to take into account factors that were not measured in the water collection sites but that could be related to their geographical localization.

In bivariate analysis, a higher probability of presence of anopheline larvae was found for water collection sites that were temporary and those with perimeter <20 m, with surface vegetation covering less than 20% of the total area, with a temperature >= 30°C, or with a co-occurrence of *Culicinae *larvae. Anopheline larvae were also mainly found in ditches, puddles (all of them being temporary) and swamps, ponds or lakes (about half of them being temporary), highlighting the importance of temporary water collections in larval presence. In multivariate analysis, habitat type and presence of *Culicinae *larvae remained significant.

Higher larval densities were associated with water temperature >= 30°C, surface vegetation covering less than 20% of the total surface area, co-occurrence of *Culicinae *larvae and habitat type. All four parameters remained significant in multivariate analysis.

These results are consistent with the known preference of An. *gambiae s.l*. for breeding in temporary pools [27,41]. Co-occurrence of *Anopheles *and *Culicinae *larvae was previously reported in the literature [11]. Low-floating vegetation was also previously found as a determinant of the presence of anopheline larvae [11,42].

The other physico-chemical parameters measured in the water collection sites were not significantly associated either with the occurrence or the abundance of larvae. Concerning turbidity, conflicting results were found in previous studies. Higher turbidity has been associated positively [42] or negatively [7] with the presence of anopheline larvae. In Dakar, Robert *et al*. found a preference for breeding in clear water in the "céanes" [28]. As turbidity can be an indicator of particulate matter in suspension that could be food for larvae or polluting agents, its effect remains unclear. Even though water collection sites exposed to sunlight are known to provide breeding places for *An. gambiae s.l*. [27,41], the present study could not show any association with this parameter, probably because of a lack of contrast (*i.e*., very few water collection sites with no sunlight exposure) and the consequent lack of analysis power.

The presence of predator fish was not associated either with a lower probability of larvae or lower larval density. Finally, the period of prospection was associated neither with the presence of larvae nor with larval density.

One possible limitation of the data interpretation may have been the use of the dipping method, which could have failed to provide repetitive measures of larval densities even though the sampling location and the person collecting the samples were consistent throughout the study. The presence/absence of larvae was probably recorded correctly with this technique, especially for small habitats.

### Adaptation to urban settings

The data recorded from water collection sites did not aim to measure pollution but anopheline larvae were sampled in bodies of water that, based on visual examination, appeared polluted. In urbanized environments such as the ones in the present study areas, it cannot be excluded that *Anopheles *can adapt to new conditions, as was previously shown in some studies. In Accra, *An. gambiae s.l*. evolution over a few decades led to a rise in breeding in domestic water and polluted water [6]. In Dar Es Salaam, *An. gambiae s.l*. bred in organically polluted habitats [7]. *Anopheles gambiae s.s*. larvae have been found in water polluted with heavy metals and oil in Lagos [8].

### Urban agriculture

The importance of urban agricultural activity on malaria has been reported in several African cities, such as in Côte d'Ivoire and Ghana [10], where irrigation led to the emergence of larval habitats [10,11] and higher malaria prevalence [12,13]. It has been suggested that irrigated vegetable fields around a French military camp in Abidjan could have been the source of the unexpectedly high number of adult *Anopheles *caught there [43]. In other cities such as Malindi in Kenya, no relationship has been found between household-level urban agriculture and the occurrence of bodies of water [44].

Two of the areas in the present study (Pikine and Yarakh) sustained urban agricultural activities, but no irrigation systems were in place. Watering was done manually every morning, with water coming from the "céanes." In both areas, infected adult *Anopheles *were caught on human bait. This was consistent with results in 2005–2006 where infected *Anopheles *have been caught in Dakar (district of Ouakam), close to market gardens [26].

Among the studied "céanes," only three (37%) harboured larvae and the larval densities were very low. These results were consistent with those reported by Robert *et al *in 1998, in which only 33% of 48 "céanes" harboured anopheline larvae, with low densities [28]. Even though the presence of mosquito-eating fishes was not significantly associated with the presence/absence of larvae and larval density in the statistical analysis, these low larval densities could be partly linked to the systematic presence of larvivorous fishes, only allowing larvae to grow if they are hidden in the floating vegetation. These fishes were introduced in Dakar in the 1930s for larval control and their presence in the wells is recommended by the National Hygiene Service. In 1998, Awono-Ambéné *et al *[29] confirmed their utility, indicating that predation in the "céanes" was probably mainly due to fishes (*Gambusia *and *Tilapia*). Other factors which were not measured in this study, such as the use of pesticides, could also lead to low larval density in the "céanes". Furthermore, it is possible that in the rainy season temporary breeding sites were more attractive for breeding than the "céanes," as higher larval densities in the céanes were recorded at the end of the dry season but not during the rainy season [29].

In Pikine, some temporary and permanent breeding sites which were not linked to market-gardening were present, so it was difficult to measure the link between the agricultural activity and the adult *Anopheles *density. In contrast, water collections were highly related to urban agriculture in Yarakh but larval densities were low as previously described in Dakar [28], so it is probable that market-gardens provided resting sites to *Anopheles *rather than increased number of breeding sites, as was previously demonstrated in Ghana [45].

### Spatial scale of malaria transmission

In the present study, a very close parallel was found between larval density index and adult densities. In six out of ten areas, it was possible to superimpose temporal variations in larval and adult densities. This correlation is consistent with the hypothesis of a malaria transmission system that is contained within the limits of the study areas. The larval habitats available in each area could be sources of adult mosquitoes, or at least were representative of the habitats available in a larger area providing the adult specimens caught on human bait. The parallel between larval density index and adult densities was not found by taking into account all areas together, as no information on population and building densities were available to balance the associations.

The present results were consistent with the low dispersion (<300 metres) of *Anopheles *from their breeding habitats. In rural areas, dispersion can reach several kilometres, and it is highly reduced in urban settings, due to the high density of houses and the proximity of readily available hosts for blood meals. Several studies have highlighted the low dispersion of *Anopheles *in urban settings, its consequences in terms of heterogeneity of malaria transmission levels and its implications for the incidence of clinical malaria. In Pikine, a gradient of *Anopheles *density and malaria prevalence was shown on a 910-metre transect, going from the marshland to the city centre. Most of the *An. arabiensis *were caught at <285 m from the marshland [14].

Similar examples also exist in other African cities. In Ouagadougou, Burkina-Faso, most of the *An. gambiae s.l*. females were collected within 300 m of the breeding sites located along a water reservoir [46] and the *P. falciparum *infections where concentrated in the human population living within 200 m of the hydrographic network [47]. In two cities in Cameroon, *Anopheles *densities recorded on hilly slopes (around 40 m high) were zero at 200 and 250 m from the swampy valleys where the breeding sites are concentrated [48]. In Edea, also in Cameroon, the EIR varied from 0 to 86 infective bites per person per year between houses within 200 m of each other, depending on their proximity to the breeding sites [49]. In Brazzaville, Congo, great heterogeneity of transmission was recorded between districts, ranging from more than 100 infective bites per person per year to less than one infective bite per person every three years [50]. Maps of malaria transmission intensity in Brazzaville showed that districts with very different malaria risk levels could be adjacent to each other [51]. In Uganda, proximity to the breeding habitats has been recognized as a risk factor for clinical malaria episodes at scales of a few hundred metres [15].

The method used in the present study for the measurement of larval densities accounted for all larval stages including pupae. The actual productivity of the breeding sites would have been better estimated by accounting for stage IV and pupae only, as the dynamics of larval mortality could differ depending on the type of breeding habitat [52]. To obtain such estimates, the larval sampling effort should be much greater. However, this is not a limitation in the present study, as the larval and adult densities were compared temporally within each study area. Since the figures were relative and not absolute, the comparisons were valid.

### Temporal dynamics of malaria transmission

A very close parallel was found between the adult density and the larval density index but not between the adult density and the raw larval density if the latest was not adjusted on breeding sites surfaces. As water collection surfaces are driven by rainfall amounts and frequency of rainfall events, relationship between meteorological data and *Anopheles *larval and adult densities should be further investigated.

### Implications for malaria control

Since the beginning of the 21st century, there has been renewed interest in larval control as part of an integrated malaria control strategy (including ITNs, indoors residual insecticide spraying and health care access) [53]. In urban settings, the human population density relative to the number of breeding sites is very high, so larval control could be effective [54].

Focusing on larval control in African urban areas could lead to satisfactory results, as was the case in Palestine/Israel, Italy and the United States, where the modification or elimination of aquatic habitats was applied extensively and contributed significantly to the eradication of malaria transmission, especially in urban settings [55]. Regarding the *An. gambiae *complex, several reports of successful control efforts have been reported. In Ethiopia, environmental management led to a 49% reduction in *An*. *arabiensis *adult density [56]. In the city of Dar Es Salaam, the killing of larvae succeeded in significantly reducing malaria transmission and morbidity [57]. In Djibouti City, malaria has been controlled by larval control using larvivorous fishes [58]. In Brazil, *An*. *arabiensis *invaded the North-East region in the thirties and led to a ten-year malaria epidemic [59]. Focus on larval control made it possible to eradicate the vector and the disease [60] from the area while the proportion of temporary breeding sites was high, just as it is in Dakar.

In areas where transmission is low or moderate, focusing malaria control activities in limited areas should greatly improve their efficacy and their cost-effectiveness [16]. Thus, a good knowledge of mosquito dynamics and of the ecological requirements leading to the presence of breeding sites is crucial. A deep understanding could even help to target larval control to the most productive habitats, thus enhancing the efficacy of control [61]. The present study has shown that transmission in Dakar is highly focal, at scales of a few hundred metres, and that there is a high degree of correlation between larval and adult densities within each area. Furthermore, transmission is temporally focal and lasts only a few weeks. Even if productive habitats in Dakar show a great variety, they could be spatially and temporally identified; in this context, malaria control in Dakar could benefit from larval management.

## Conclusion

In Dakar, malaria transmission exists and is highly focal. In order to spatially focus malaria control in the areas that are at greater risk, precise mapping of malaria transmission levels should be conducted. With new technologies such as Geographic Information Systems and Remote Sensing, associated with entomological and epidemiological work, the possibility of mapping the malaria risk in Dakar exists and should be further explored.

## Competing interests

The authors declare that they have no competing interests.

## Authors' contributions

VM was responsible for the study design, supervision of data collection, analysis, interpretation, production of the final manuscript and revisions. LG contributed to the supervision of data collection, data analysis, interpretation and production of the final manuscript. CV contributed to the study design, analysis and interpretation. FJ contributed to the data analysis. SB contributed to the data analysis. JPL contributed to overall scientific management, analysis, interpretation, preparation of the final manuscript and revisions. JFT contributed to overall scientific management, analysis, interpretation, preparation of the final manuscript and revisions. CR was responsible for overall scientific management, analysis, interpretation, preparation of the final manuscript and revisions. FP was responsible for overall scientific management, analysis, interpretation, preparation of the final manuscript and revisions. All authors read and approved the final manuscript.
